# Supplementary material for: Association between serum mineral concentrations and gastrointestinal parasite burden in zebu cattle accessing ‘hora’ mineral water in southwestern Ethiopia
Source: PLoS One. 2025 Aug 18;20(8):e0329812. doi: 10.1371/journal.pone.0329812 (PMC12360600; doi:10.1371/journal.pone.0329812)
Supplement: S1 File — Lists of tables of descriptive statistics of analyzed data. (DOCX) [file pone.0329812.s001.docx]

**Appendix**

**Appendix 1: Descriptive statistics of serum mineral status of zebu cattle**

|  | | Mean | Std. Error | Minimum | Maximum |  |  |  |
| --- | --- | --- | --- | --- | --- | --- | --- | --- |
|  |  |  |  |  |  | CV | **χ2** | p-value |
| Ca | Dabo | 87.6260 | 7.57194 | 54.84 | 114.78 | 27.3 |  |  |
|  | Gechi | 62.4800 | 5.01186 | 34.56 | 82.76 | 25.4 |  |  |
|  | Bedele | 66.6020 | 4.59228 | 41.64 | 88.20 | 21.8 |  |  |
|  | Borecha | 220.0900 | 22.24578 | 111.00 | 298.42 | 32.0 |  |  |
|  | Total | 109.1995 | 11.91453 | 34.56 | 298.42 | 69.0 | 24.187 | <0.001 |
| Mg | Dabo | 20.3060 | 1.53333 | 13.77 | 26.35 | 23.9 |  |  |
|  | Gechi | 14.2880 | .64419 | 11.25 | 17.85 | 14.3 |  |  |
|  | Bedele | 15.3790 | .90022 | 10.22 | 18.88 | 18.5 |  |  |
|  | Borecha | 41.1560 | 3.91046 | 24.24 | 67.98 | 30.0 |  |  |
|  | Total | 22.7822 | 2.02627 | 10.22 | 67.98 | 56.3 | 26.787 | <0.001 |
| Na | Dabo | 2496.6360 | 79.46656 | 1995.36 | 2790.66 | 10.1 |  |  |
|  | Gechi | 2644.8360 | 118.34642 | 1832.16 | 3191.76 | 14.1 |  |  |
|  | Bedele | 2491.4100 | 188.39109 | 1310.04 | 2911.98 | 23.9 |  |  |
|  | Borecha | 2514.8220 | 145.42983 | 1806.24 | 3454.14 | 18.3 |  |  |
|  | Total | 2536.9260 | 67.39117 | 1310.04 | 3454.14 | 16.0 | 2.595 | 0.458 |
| K | Dabo | 233.7020 | 9.87098 | 192.12 | 282.82 | 13.4 |  |  |
|  | Gechi | 255.0000 | 9.05288 | 187.62 | 282.36 | 11.2 |  |  |
|  | Bedele | 212.5020 | 11.38501 | 154.08 | 251.76 | 16.9 |  |  |
|  | Borecha | 257.8320 | 9.31696 | 203.40 | 302.28 | 11.4 |  |  |
|  | Total | 239.7590 | 5.60499 | 154.08 | 302.28 | 14.8 | 10.279 | 0.016 |
| P | Dabo | 86.1820 | 5.41504 | 55.08 | 102.66 | 19.9 |  |  |
|  | Gechi | 86.2080 | 4.12250 | 63.66 | 99.78 | 15.1 |  |  |
|  | Bedele | 78.9780 | 7.91456 | 34.20 | 109.68 | 31.7 |  |  |
|  | Borecha | 114.5040 | 9.36847 | 79.08 | 159.78 | 25.9 |  |  |
|  | Total | 91.4680 | 4.01344 | 34.20 | 159.78 | 27.8 | 5.732 | 0.125 |
| S | Dabo | 817.6260 | 28.93504 | 578.10 | 881.16 | 11.2 |  |  |
|  | Gechi | 840.9960 | 61.90715 | 555.48 | 1174.08 | 23.3 |  |  |
|  | Bedele | 767.7720 | 73.10916 | 314.04 | 963.96 | 30.1 |  |  |
|  | Borecha | 788.2090 | 37.89421 | 589.62 | 1006.20 | 15.2 |  |  |
|  | Total | 803.6508 | 26.08806 | 314.04 | 1174.08 | 20.5 | 0.999 | 0.801 |
| Fe | Dabo | 5.0040 | .62287 | 2.28 | 8.46 | 39.4 |  |  |
|  | Gechi | 3.1320 | .19028 | 2.22 | 3.92 | 19.2 |  |  |
|  | Bedele | 2.4810 | .19587 | 1.52 | 3.25 | 25.0 |  |  |
|  | Borecha | 22.1960 | 1.17952 | 16.84 | 26.88 | 16.8 |  |  |
|  | Total | 8.2033 | 1.34254 | 1.52 | 26.88 | 104.0 | 29.405 | <0.001 |
| Mn | Dabo | 13.2490 | .42464 | 11.68 | 15.80 | 10.1 |  |  |
|  | Gechi | 4.2490 | .42464 | 2.68 | 6.80 | 31.6 |  |  |
|  | Bedele | 5.3110 | .39832 | 3.68 | 7.71 | 23.7 |  |  |
|  | Borecha | 8.2490 | .42464 | 6.68 | 10.80 | 16.3 |  |  |
|  | Total | 7.7645 | .59371 | 2.68 | 15.80 | 48.4 | 32.863 | <0.001 |
| Cu | Dabo | .5440 | .02349 | .42 | .66 | 13.7 |  |  |
|  | Gechi | .5660 | .03807 | .42 | .78 | 21.3 |  |  |
|  | Bedele | .3970 | .04629 | .18 | .66 | 36.9 |  |  |
|  | Borecha | 1.7940 | .21634 | 1.14 | 2.94 | 38.1 |  |  |
|  | Total | .8253 | .10521 | .18 | 2.94 | 80.6 | 26.757 | <0.001 |
| Zn | Dabo | 6.5910 | .21798 | 5.25 | 7.66 | 10.5 |  |  |
|  | Gechi | 1.6680 | .15271 | 1.11 | 2.33 | 29.0 |  |  |
|  | Bedele | 2.0650 | .12859 | 1.44 | 2.56 | 19.7 |  |  |
|  | Borecha | 3.5910 | .21798 | 2.25 | 4.66 | 19.2 |  |  |
|  | Total | 3.4788 | .32215 | 1.11 | 7.66 | 58.6 | 32.741 | <0.001 |
| Mo | Dabo | 3.4260 | .30784 | 2.04 | 5.64 | 28.4 |  |  |
|  | Gechi | 3.6660 | .23570 | 2.52 | 5.28 | 20.3 |  |  |
|  | Bedele | 4.0200 | .36011 | 2.28 | 5.76 | 28.3 |  |  |
|  | Borecha | 3.3040 | .13869 | 2.46 | 4.08 | 13.3 |  |  |
|  | Total | 3.6040 | .13848 | 2.04 | 5.76 | 24.3 | 3.231 | 0.357 |

**Appendix 2: Mean serum mineral concentrations of zebu (*Bos indicus*) cattle grazing around and accessing *hora* mineral water in southwestern Ethiopia.**

| **Minerals** | Mean± SE | Range | Diagnostic criteria |  | |
| --- | --- | --- | --- | --- | --- |
|  |  |  | Deprived^[[1]](#footnote-1)^ | Dermau et al^[[2]](#footnote-2)^ | |
| Ca, mg/L | 109.2±11.9 | 34.6-298.4 | 80 mg/L | 5.4±1.2 mmol/l | 216.43 mg/L |
| Mg, mg/L | 22.8±2.2 | 10.2-67.9 | 17.0 mg/L | 1.9±0.2 mmol/l | 46.20 mg/L |
| Na, mg/L | 2536.9±67.4 | 1310.1-3454.1 | 140 mg/L | 131±6 mmol/l | 3016.69 mg/L |
| K, mg/L | 239.8±5.6 | 154.1-302.3 | 97.7 mg/L | 4.4±0.8 mmol/l | 172.04 mg/L |
| P, mg/L | 91.5±4.0 | 34.2-159.8 | 46.5 mg/L | 3.3±0.6 mmol/l | 102.23 mg/L |
| S, mg/L | 803.6±26.1 | 314.0-1174.1 | - | 37±4 mmol/l | 1187.59 mg/L |
| Fe, mg/L | 8.2±1.3 | 1.5-26.9 | 1.0 mg/L | 143±133 μmol/l | 7.99 mg/L. |
| Mn, mg/L | 7.8±0.6 | 2.7-15.8 | 1.1 mg/L | - | - |
| Cu, mg/L | 0.8±0.1 | 0.2-2.9 | 1.2 mg/L | 6.0±4.3 μmol/l | 381.30 mg/L |
| Zn, mg/L | 3.5±0.3 | 1.1-7.7 | 0.6 mg/l | 46±17 μmol/l | 3.01 mg/L |
| Mo, mg/L | 3.6±0.1 | 2.0-5.8 | - | 37±6 μmol/l | 3.55 mg/L |

SE = standard error

**Appendix 3: Descriptive statistics of mineral concentration of hora**

|  | | Mean | Std. Deviation | Std. Error | Minimum | Maximum |
| --- | --- | --- | --- | --- | --- | --- |
|  |  |  |  |  |  |  |
| Ca | Dabo | 16.2103 | 1.11881 | .64595 | 14.92 | 16.89 |
|  | Bedele | 48.8870 | 1.49045 | .86051 | 47.44 | 50.41 |
|  | Borecha | 7.0497 | .63564 | .36698 | 6.55 | 7.77 |
|  | Gechi | 8.1793 | .69001 | .39838 | 7.60 | 8.94 |
|  | Total | 20.0816 | 17.77998 | 5.13264 | 6.55 | 50.41 |
| Mg | Dabo | 17.0893 | .45917 | .26510 | 16.67 | 17.58 |
|  | Bedele | 32.3593 | .72579 | .41903 | 31.55 | 32.94 |
|  | Borecha | .9037 | .10291 | .05941 | .79 | .99 |
|  | Gechi | 1.9990 | .12663 | .07311 | 1.86 | 2.09 |
|  | Total | 13.0878 | 13.40958 | 3.87101 | .79 | 32.94 |
| Na | Dabo | 734.3443 | 6.12579 | 3.53672 | 728.11 | 740.35 |
|  | Bedele | 551.4020 | 8.09080 | 4.67123 | 542.06 | 556.15 |
|  | Borecha | 351.1200 | 2.52335 | 1.45686 | 349.14 | 353.96 |
|  | Gechi | 382.6803 | 3.93379 | 2.27118 | 378.17 | 385.39 |
|  | Total | 504.8867 | 159.66695 | 46.09188 | 349.14 | 740.35 |
| K | Dabo | 21.8977 | .55268 | .31909 | 21.41 | 22.50 |
|  | Bedele | 26.4037 | .56331 | .32523 | 25.79 | 26.90 |
|  | Borecha | 5.7990 | .56592 | .32674 | 5.45 | 6.45 |
|  | Gechi | 4.9607 | .54446 | .31435 | 4.33 | 5.28 |
|  | Total | 14.7653 | 9.95911 | 2.87495 | 4.33 | 26.90 |
| P | Dabo | 2.4383 | .04245 | .02451 | 2.40 | 2.49 |
|  | Bedele | 2.5787 | .08317 | .04802 | 2.48 | 2.64 |
|  | Borecha | 1.6587 | .05705 | .03294 | 1.60 | 1.72 |
|  | Gechi | 1.7280 | .02078 | .01200 | 1.70 | 1.74 |
|  | Total | 2.1009 | .43223 | .12477 | 1.60 | 2.64 |
| S | Dabo | 29.5527 | 1.02218 | .59016 | 28.59 | 30.62 |
|  | Bedele | 17.2470 | .36250 | .20929 | 16.90 | 17.62 |
|  | Borecha | .1520 | .00954 | .00551 | .15 | .16 |
|  | Gechi | 2.5890 | .20922 | .12079 | 2.45 | 2.83 |
|  | Total | 12.3852 | 12.41102 | 3.58275 | .15 | 30.62 |
| Mo | Dabo | 169.6667 | 17.21434 | 9.93870 | 150.00 | 182.00 |
|  | Bedele | 147.0000 | 6.00000 | 3.46410 | 141.00 | 153.00 |
|  | Borecha | 145.6667 | 8.96289 | 5.17472 | 140.00 | 156.00 |
|  | Gechi | 115.0000 | 1.00000 | .57735 | 114.00 | 116.00 |
|  | Total | 144.3333 | 22.07563 | 6.37268 | 114.00 | 182.00 |
| Fe | Dabo | 463.0000 | 5.29150 | 3.05505 | 459.00 | 469.00 |
|  | Bedele | 1585.3333 | 4.04145 | 2.33333 | 1583.00 | 1590.00 |
|  | Borecha | 1154.0000 | 17.05872 | 9.84886 | 1135.00 | 1168.00 |
|  | Gechi | 884.3333 | 11.71893 | 6.76593 | 871.00 | 893.00 |
|  | Total | 1021.6667 | 426.35332 | 123.07760 | 459.00 | 1590.00 |
| Mn | Dabo | 3.1667 | .28868 | .16667 | 3.00 | 3.50 |
|  | Bedele | 53.6667 | 1.52753 | .88192 | 52.00 | 55.00 |
|  | Borecha | 18.0000 | 1.00000 | .57735 | 17.00 | 19.00 |
|  | Gechi | 19.0000 | 1.00000 | .57735 | 18.00 | 20.00 |
|  | Total | 23.4583 | 19.37836 | 5.59405 | 3.00 | 55.00 |
| Cu | Dabo | 51.6667 | 1.52753 | .88192 | 50.00 | 53.00 |
|  | Bedele | 45.9533 | .30665 | .17704 | 45.60 | 46.15 |
|  | Borecha | 45.0000 | 1.73205 | 1.00000 | 43.00 | 46.00 |
|  | Gechi | 48.0000 | 1.00000 | .57735 | 47.00 | 49.00 |
|  | Total | 47.6550 | 2.88139 | .83178 | 43.00 | 53.00 |
| Zn | Dabo | 85.3333 | .57735 | .33333 | 85.00 | 86.00 |
|  | Bedele | 131.6667 | .57735 | .33333 | 131.00 | 132.00 |
|  | Borecha | 77.6667 | 2.51661 | 1.45297 | 75.00 | 80.00 |
|  | Gechi | 114.6667 | 1.52753 | .88192 | 113.00 | 116.00 |
|  | Total | 102.3333 | 22.86058 | 6.59928 | 75.00 | 132.00 |
| Se | Dabo | 15.0000 | 1.00000 | .57735 | 14.00 | 16.00 |
|  | Bedele | 32.0000 | 1.73205 | 1.00000 | 31.00 | 34.00 |
|  | Borecha | 14.3333 | .57735 | .33333 | 14.00 | 15.00 |
|  | Gechi | 24.0000 | 1.00000 | .57735 | 23.00 | 25.00 |
|  | Total | 21.3333 | 7.63167 | 2.20307 | 14.00 | 34.00 |

1. Upper marginal band for diagnostic criteria indicating a probable deprivation risk in *Bos taurus* cattle according to Suttle (2010) [↑](#footnote-ref-1)
2. Dermauw et al. (2013): plasma macrominerals in *Bos indicus* cattle grazing in a comparable environment [↑](#footnote-ref-2)
