# Supplementary material for: Association between serum mineral concentrations and gastrointestinal parasite burden in zebu cattle accessing ‘hora’ mineral water in southwestern Ethiopia
Source: PLoS One. 2025 Aug 18;20(8):e0329812. doi: 10.1371/journal.pone.0329812 (PMC12360600; doi:10.1371/journal.pone.0329812)
Supplement: Attachment — Submitted filename: Response_to_Reviewers.docx [file pone.0329812.s005.docx]

**Authors’ response to editor and reviewers’ comments**

Thank you very much for taking the time to assess our manuscript and giving us a chance to revise. The editors’ and reviewers’ comments and suggestions are valuable and very useful which helped us to substantially improve the quality of our paper. We have carefully read all comments and have tried our best to revise the manuscript thoroughly as per the editors’ and reviewers’ comments and suggestions, which we hope to meet with the requirements of your comments. Our reply to each comment in point-by-point way is marked in Yellow **highlight** in the response to reviewers and indicated with track change mode in revised manuscript. Once again, we would like to thank very much the respected editors and reviewers for the thoughtful and valuable comments and you precious time.

We really appreciate your help.

Sincerely,

Ashenafi Miresa Kenea

March 5, 2025

1. Please ensure that your manuscript meets PLOS ONE's style requirements, including those for file naming.

Author Response: Thank you very much for your valuable insight and comments. We prepared our revised manuscript according to PLOS ONE’s guideline.

2. Thank you for stating the following financial disclosure: “This research was supported by Jimma University College of Agriculture and Veterinary Medicine (grant number AgVmVm/M6/23) which provided funding for data collection and laboratory analysis.” Please state what role the funders took in the study. If the funders had no role, please state: "The funders had no role in study design, data collection and analysis, decision to publish, or preparation of the manuscript." If this statement is not correct you must amend it as needed. Please include this amended Role of Funder statement in your cover letter; we will change the online submission form on your behalf.

Author Response: Thank you very much for your valuable comments and reminder. As indicated in the funding statement, Jimma University College of Agriculture and Veterinary Medicine provide us financial support which we used for data collection and laboratory analysis payment. The funders had no role in study design, data collection and analysis, decision to publish, or preparation of the manuscript. We indicate the amended role of the funder in cover letter.

3. Thank you for stating the following in the Acknowledgments Section of your manuscript:
“We thank to Jimma University College of Agriculture and Veterinary Medicine for their financial support. We also extend our thanks to Buno Bedele Agriculture Office and field experts for their support during sample collection.” We note that you have provided funding information that is not currently declared in your Funding Statement. However, funding information should not appear in the Acknowledgments section or other areas of your manuscript. We will only publish funding information present in the Funding Statement section of the online submission form.
Please remove any funding-related text from the manuscript and let us know how you would like to update your Funding Statement. Currently, your Funding Statement reads as follows:
“This research was supported by Jimma University College of Agriculture and Veterinary Medicine (grant number AgVmVm/M6/23) which provided funding for data collection and laboratory analysis.” Please include your amended statements within your cover letter; we will change the online submission form on your behalf.

Author Response: Thank you very much for your valuable comments and suggestion. As indicated in the funding statement, Jimma University College of Agriculture and Veterinary Medicine provide us financial support which we used for data collection and laboratory analysis payment. However, in acknowledgment section, we want to thanks Jimma University College of Agriculture and Veterinary Medicine for their financial support and Buno Bedele Zone Agriculture office and their field experts for their technical expertise and assistance in facilitating sample collection. We clarify that Buno Bedele Zone Agricultural office are not our funding source, rather they help us technically during sample collection.

4. In the online submission form, you indicated that “The data presented in this study is available from the corresponding author upon reasonable request”. All PLOS journals now require all data underlying the findings described in their manuscript to be freely available to other researchers, either 1. In a public repository, 2. Within the manuscript itself, or 3. Uploaded as supplementary information. This policy applies to all data except where public deposition would breach compliance with the protocol approved by your research ethics board. If your data cannot be made publicly available for ethical or legal reasons (e.g., public availability would compromise patient privacy), please explain your reasons on resubmission and your exemption request will be escalated for approval.

Author Response: Thank you very much for your kind comments and suggestion. We tried to indicate the data availability statement in our previous submission (“Data is available upon request”). However, in the current submission, as per your suggestion, we provide our data and upload as supplementary information.

5. Please include your full ethics statement in the ‘Methods’ section of your manuscript file. In your statement, please include the full name of the IRB or ethics committee who approved or waived your study, as well as whether or not you obtained informed written or verbal consent. If consent was waived for your study, please include this information in your statement as well.

Author Response: Thank you very much for your comments and suggestion. We provided ethics approval statement in Material and Methods section. It is indicated in the body of manuscript and highlighted with yellow color and track change mode. Additionally, we can provide Ethical clearance letter in necessary.

6. We notice that your supplementary figures are uploaded with the file type 'Figure'. Please amend the file type to 'Supporting Information'. Please ensure that each Supporting Information file has a legend listed in the manuscript after the references list.

Author Response: Thank you very much for your comments and suggestion. In the previous submission, we mistakenly said supplementary figure, despite it is a figures used in manuscript body. Thus in the current submission we made a significant correction.

**Additional Editor Comments:**

The manuscript requires significant corrections on materials and method section as well as extensive edition on grammar, sentence structure, and language to ensure readability and adherence to the standards of journal. Please address all the comments suggested by the two reviewers.

Author Response: Thank you very much for your kind and significant comments. We rewrite the whole manuscript by including all the necessary modification. We made substantial revisions to improve the manuscript throughout. These revisions included careful review and rewriting by senior co-authors and Professor Ellen Dierenfeld, a native speaker of American English and co-author on this paper. We focused on enhancing clarity and conciseness, and we are confident the revised manuscript now meets the journal's standards for publication.

**Comments to the Author**

1. Is the manuscript technically sound, and do the data support the conclusions?

Reviewer #1: Partly

Author Response: We appreciate your positive feedback. We have carefully considered concerns regarding to sample size determination, appropriate statistical model and overall all materials and methods, and we have made significant revisions to address the concerns raised. We have also rewritten our result and discussion to improve clarity and ensure the conclusions are fully supported by the data. All modification made in the manuscript have been highlighted with yellow color and track change mode.

Reviewer #2: No

Author Response: We thank Reviewer #2 for their thorough review and critical feedback. We understand their concerns regarding the rigor of the experiments and the support for our conclusions. We have addressed these concerns by revising the Materials and Methods, Results, and Discussion sections. While we acknowledge that our sample size is small and we solely focus on specific districts, we believe that the current data, combined with the additional analyses and clarifications we have provided, strongly support our conclusions. We have also added a more detailed discussion of the limitations of our study in discussion section. We believe these changes have significantly improved the manuscript and addressed the reviewer's key concerns.

2. Has the statistical analysis been performed appropriately and rigorously?

Reviewer #1: Yes

Author Response: We thank Reviewer #1 for confirming the appropriateness of our statistical analysis. We appreciate their positive assessment.

Reviewer #2: No

Author Response: Thank you for your thorough review and critical feedback regarding the statistical analysis. We have made significant modifications to our statistical analysis. We have provided a more detailed explanation of our rationale in the Methods section to address the reviewer's concerns and ensure transparency.

3. Have the authors made all data underlying the findings in their manuscript fully available?

Reviewer #1: Yes

Author Response: Thank you for confirming that the data underlying our findings is fully available. We appreciate the reviewers' positive assessment of our data availability. As stated in our Data Availability Statement, all data are freely accessible with supporting information.

Reviewer #2: Yes

Author Response: Thank you for confirming that the data underlying our findings is fully available. We appreciate the reviewers' positive assessment of our data availability. As stated in our Data Availability Statement, all data are freely accessible with supporting information.

4. Is the manuscript presented in an intelligible fashion and written in standard English?
PLOS ONE does not copyedit accepted manuscripts, so the language in submitted articles must be clear, correct, and unambiguous. Any typographical or grammatical errors should be corrected at revision, so please note any specific errors here.

Reviewer #1: No

Author Response: We thank Reviewer #1 for their feedback. While they did not provide specific examples of language issues, we acknowledge the reviewers' concerns regarding clarity and English language. We have therefore made substantial revisions to improve the manuscript throughout. These revisions included careful review and rewriting by senior co-authors and Professor Ellen Dierenfeld, a native speaker of American English and co-author on this paper. We focused on enhancing clarity and conciseness, and we are confident the revised manuscript now meets the journal's standards for publication.

Reviewer #2: No

Author Response: We thank Reviewer #2 for their feedback. Although specific examples of language issues were not provided, we have taken the reviewer's comments seriously and have made substantial revisions to improve the language and clarity of the manuscript. We have clarified the terminology used throughout the manuscript and ensured consistency in style and formatting.

**5. Review Comments to the Author**

**Reviewer #1:** The authors have examined only 45 animals from each district. The manuscript should provide a clear explanation of how this sample size was determined and the basis for selecting this specific number. Include statistical or logistical reasoning to justify the representativeness of the sample size.

Author Response: We thank Reviewer #1 for their comment regarding sample size determination. Our sample size of 45 animals per district was determined by considering the recommendations for mineral status assessment (Ensley 2020) and endoparasite monitoring (Maurizio et al., 2021). Given that our study assessed both mineral status and endoparasites, we selected a sample size that fell within the upper end of these recommended ranges to ensure adequate representation for both parameters. We felt that 45 animals per district provided a balance between statistical robustness and the logistical constraints of our study.

**Reviewer #1:** The authors used 100x magnification to examine the eggs. Provide a justification for choosing this magnification instead of 10x or 40x, which are commonly used for egg examination. Clarify why 100x was deemed necessary and how it improved the accuracy or visibility of the eggs.

Author Response: We thank Reviewer #1 for their comment regarding magnification. While 10x and 40x magnifications are commonly used for general egg/oocyst identification, 100x magnification was chosen for better visualization of key morphological features of the egg*.* This improved the accuracy of identification compared to what would have been possible at lower magnifications.

**Reviewer #1:** Add suitable references to support statements made in lines 92-102 and 124-134. Ensure that these references are relevant, up-to-date, and from credible scientific sources to strengthen the manuscript’s credibility. Ensure that all scientific names throughout the text are italicized, including those mentioned in Figure 1. This is a standard convention in scientific writing.

Author Response: Thank you very much for your suggestion and comment. We accepted your comments and add all relevant and up-to-date reference to support sour statement and strength the manuscript credibility. All modification was shown with highlighted yellow color and track change mode in the text. We also italicized all scientific name in our manuscript.

**Reviewer #1:** The manuscript does not thoroughly discuss its limitations. Include a section addressing potential limitations such as: Sample size constraints.

Author Response: We acknowledge the reviewers concern regarding sample size constraints. The sample size for this study was determined based on recommendations of (Ensley 2020) serum mineral analysis and (Maurizio et al., 2021) and endoparasites monitoring. These studies suggested that a sample size of 7-20 animals per the herd would be sufficient to detect a meaningful correlation between mineral status and parasite burden. Based on these recommendations, we aimed to collect a total of 180 samples from four different districts. While this sample size allowed us to observe a statistically significant correlation, we recognize that it may still be considered a limitation. Furthermore, we include the significant limitation of our study under discussion section.

**Reviewer #1:** Reliance on specific districts and whether the findings can be generalized. Other potential health and environmental factors that might influence parasite prevalence but were not considered in the study.

Author Response: Thank you for your valuable comments regarding the limitation of our study. We acknowledge that our reliance on specific districts and the exclusion of other potential health and environmental factors limit the generalizability of our findings. Our focus on these specific districts was driven by the unique presence of hora mineral water, which is geographically restricted and central to our research question. As hora is a mineral supplement used by local cattle owners’ in these areas, our study specifically aimed to investigate the correlation between hora-influenced serum mineral status and gastrointestinal parasite burden in this target animal group. We recognize that other factors, could also influence parasite prevalence. While these factors were not directly considered in this study, we acknowledge that they could potentially contribute to the observed parasite burden and may influence the generalizability of our findings to other regions or cattle populations. We have now added a more detailed discussion of these limitations, including their potential impact on the interpretation of our results, to the limitation section.

**Reviewer #1:** Some parts of the discussion lack clarity and depth. Revise this section to ensure that the results are interpreted comprehensively and placed in the context of existing literature. Provide more detailed insights into the implications of the findings. The manuscript requires significant editing for grammar, sentence structure, and language to ensure readability and adherence to the standards of journal. Additionally, ensure consistency in formatting across all sections.

Author Response: Thank you for your valuable feedback. We have made significant revisions to the Discussion section to address the concerns regarding clarity, depth, and context. We have expanded our interpretation of the observed correlation between and parasite load. We integrate our finding with existing literature to compare and contrast our studies with previous related studies. We have expanded the discussion of the implications of our findings animal health management, disease prevention strategies, and future research directions. In addition to the revisions to the Discussion section, we have also thoroughly edited the entire manuscript for grammar, sentence structure, and language to improve readability and ensure adherence to journal standards. We have also carefully reviewed the formatting throughout the manuscript to ensure consistency.

**Reviewer #2:** “Correlation of mineral status with gastrointestinal parasite burden in zebu (Bos indicus) cattle grazing around natural mineral water (hora) in southwestern Ethiopia” aims to investigate the correlation between serum mineral status and gastrointestinal parasite burden in zebu cattle grazing around and accessing natural mineral water sources in southwestern Ethiopia. However, there are several issues with the manuscript that made me reject it for publication. The main ones are related to the material and methods. There are a series of non-controlled variables that can affect the results, which basically compare regions. For instance, lack of information and collection criteria about breed, age and physiological status from the animal side; pasture availability, forage composition, and “hora” water composition from the nutrition side. Definitely, methods are not appropriate to achieve the goals. Blood was left to coagulate. Hemolysis of serum samples is recognized to be the leading cause of preanalytical errors in clinical laboratories because it can significantly affect the mineral status of cattle serum, leading to inaccurate mineral profile assessments (doi: 10.3390/ani11123336). Also, being qualitative, parasitological analysis is not suitable to establish any correlation with nutrition because it indicates the prevalence and not the parasite burden. The manuscript looks like more an attempt to assess mineral status and gastrointestinal parasite in zebu cattle in southwestern Ethiopia than a research paper to me. With the right methodologies, it could be a research note. Finally, it is inaccurate to analyze correlations with numerous uncontrollable variables.

Author Response: Thank you very much for your thorough review and constructive criticism. We acknowledge the reviewer's concerns regarding the limitations of our study, particularly the potential influence of uncontrolled variables on our results. We have carefully considered these comments. While we cannot fully address all concerns within the scope of this study, we have made the following revisions to acknowledge the limitations and clarify the study's focus.

**Animal-Related Variables (Breed, Age, and Physiological Status):** We have now included more detailed information about the animals used in the study. We used sexually matured male and dry female (in the age range of 5-7 years) of Zebu cattle (horro breed) which are found in the study area to minimize variability related to age and physiological status. We have also added a discussion of how these factors might have influenced our results and acknowledge that future studies with more controlled animal populations would be beneficial.

**Nutrition-Related Variables (Pasture, Forage, Hora Composition):** We acknowledge the reviewer's concern regarding the variability in pasture availability, forage composition, and hora water composition. We have laboratory result data of hora mineral water, which is the main target of our study. We provided our laboratory data of mineral concentration of hora mineral water which is used as mineral supplement for free ranging cattle in the study area. Despite it is important to know the mineral composition and nutritive value of forage and pasture used as a major livestock feed in the study area, due to time and financial constraint, we were unable to collect comprehensive data on these variables. We acknowledge that variations in these factors could have influenced our results, and we have discussed this limitation in the manuscript.

**Hemolysis of Serum Samples:** We thank the reviewer for highlighting the potential impact of hemolysis on serum mineral analysis. We took different precautions including handle the blood sample gently to avoid mechanical stress that can cause hemolysis and we placed the blood sample at 4°C for clotting overnight to slow down the clotting process and minimize hemolysis. Despite we took different precautions, we have added a discussion of this potential limitation to the manuscript.

**Parasitological Analysis:** We thank Reviewer #2 for their thorough review and constructive criticism. We appreciate the reviewer's point about the importance of quantitative data for correlation analysis. We would like to clarify that our parasitological analysis included Egg Counts Per Gram (EPG), which is a quantitative measure of parasite burden. We have now clarified this in the Materials and Methods section and have ensured that our analyses and interpretations are based on this quantitative data. While we also performed qualitative assessments to identify parasite species, our correlation analyses focused exclusively on the quantitative EPG data. Given that we have quantitative EPG data, we believe that correlation analysis is appropriate to assess the relationship between mineral status and parasite burden. We have clarified in the manuscript that our correlation analyses are based on EPG values.

We acknowledge the reviewer's concern that the manuscript reads more like an assessment than a research paper. We believe this perception stems from the limitations inherent in field studies conducted under natural grazing conditions, where controlling all variables is challenging. However, we have made revisions to clarify the study's scope and emphasize its contribution as valuable preliminary data.
