# Supplementary material for: Association between serum mineral concentrations and gastrointestinal parasite burden in zebu cattle accessing ‘hora’ mineral water in southwestern Ethiopia
Source: PLoS One. 2025 Aug 18;20(8):e0329812. doi: 10.1371/journal.pone.0329812 (PMC12360600; doi:10.1371/journal.pone.0329812)
Supplement: Attachment — Submitted filename: Comments_PLoS One.docx [file pone.0329812.s006.docx]

**Comments**

The paper describes the effects of drinking mineral water on GI parasites. It is well composed and well organized paper. The language and coherence of the paper are commendable. However, there are flaws and issues of clarities in design, sampling technique, coprological methods, and results section as addressed below. Therefore, the paper needs revision and clarification before considering for publication.

**Specific comments**

**Materials and Methods**

*There is a design weakness:*

1. The sites selected for the study were all having access to mineral water (hora). To explore the effect of drinking mineral water on the GIT parasites burden or prevalence as indicated in the title and objectives, comparison sites, which do not have access to mineral water should be selected as control.

2. The comparison was made within the same group, which have access to mineral water. This does not detect any correlation between mineral status and GIT burden

3. It seems that the animals included in the study had low EPG (Table 3). It is difficult to discern if this low EPG is due to the effects of mineral water or lower parasite in the pasture in the absence of controls

**It is better to use map to depict the study sites**

*Sampling methods:*

1. How did the authors randomize their selection? There is no individual animal identification system in Ethiopia, which is requisite for randomization

2. The authors did not mention anything about the welfare issues in relation to handling of animals during sample collection (fecal and blood samples)

*Coprological techniques*:

1. How long did the fecal suspension sediment? This was not described by the authors

2. Was quantitative analysis done for trematodes only? According to the description of the authors on page 5, it seems that quantitative assay was done only for trematodes

*Data analysis:*

The authors need to explicitly explain how the association between the EPG and serum mineral status was done using chi-squared test. Did they dichotomize the EPG and or the mineral concentrations? In the results section they reported Pearson correlation coefficient not Pearson Chi-square test.

**Results:**

1. The authors reported the results to species level for parasites and to the genus level for others (figure 1). The authors did not perform culture. So, how did they do identification to species and genus level based flotation and sedimentation alone?

2. The districts are dominantly found in mid to lowland agro ecology and similar weather conditions (from the study site description). Hence, how did the authors identify nematodes such as *Nematodirus* and *Ostertagia*? Their identification lacks reliability.

**Discussion**:

The authors need to discuss the significance their findings (provide explicit and implicit explanations) before making comparisons with previous observations

On page 10, the authors provided “*The mean serum macro and micro mineral status in Zebu (Bos indicus) cattle grazing around and accessing hora mineral water might be associated with the presence of hora mineral water used as a supplement in the study areas*” This explanation can be valid only if comparison is made with those cattle that graze away from hora and had lower concentrations
