# Supplementary material for: Association between serum mineral concentrations and gastrointestinal parasite burden in zebu cattle accessing ‘hora’ mineral water in southwestern Ethiopia
Source: PLoS One. 2025 Aug 18;20(8):e0329812. doi: 10.1371/journal.pone.0329812 (PMC12360600; doi:10.1371/journal.pone.0329812)
Supplement: Attachment — Submitted filename: Response_to_Reviewers_auresp_2.docx [file pone.0329812.s007.docx]

**Authors’ response to editor and reviewers’ comments**

Thank you for the opportunity to revise and resubmit our manuscript, "Correlation of serum mineral concentrations with gastrointestinal parasite burden in zebu cattle grazing around *'hora*" mineral water in southwestern Ethiopia" (PONE-D-25-01800R1). We appreciate the valuable feedback from the reviewers, which has significantly improved the quality of our manuscript. We have carefully addressed all comments raised by the academic editor and the reviewers, incorporating the suggested changes into the revised manuscript. A marked-up copy highlighting all revisions is provided as 'Revised Manuscript with Track Changes', and a clean version is provided as 'Manuscript'.

**Our detailed point-by-point responses to all comments, including those provided within the revision email text and the attached document, are presented below.**

We really appreciate your help.

Ashenafi Miresa Kenea (corresponding author)

March 5, 2025

**Academic Editor Comments:**

The reviewers have completed their evaluation of your manuscript. I encourage you to revise and resubmit your work, ensuring that all reviewer comments are thoroughly addressed. Please incorporate the feedback carefully and provide a detailed, point-by-point response that clearly outlines every change made in response to the reviewers’ suggestions. In addition, kindly correct all typographical and grammatical errors, and ensure that the manuscript is prepared in full compliance with the journal’s formatting and submission guidelines. We look forward to receiving your revised submission

Author Response: We thank the Academic Editor for this guidance. We have thoroughly reviewed our manuscript to ensure all previous reviewer comments are thoroughly addressed, as detailed in the previous revision and now in response to the current feedback. We have made a concerted effort to identify and correct all typographical and grammatical errors throughout the manuscript. We have also carefully checked and ensured full compliance with PLOS ONE’s formatting and submission guidelines, including the reference list.

**Question 1**: If the authors have adequately addressed your comments raised in a previous round of review and you feel that this manuscript is now acceptable for publication, you may indicate that here to bypass the “Comments to the Author” section, enter your conflict of interest statement in the “Confidential to Editor” section, and submit your "Accept" recommendation.

Reviewer #3: (No Response)

Author Response: We wish to thank Reviewer #3 for their time and constructive evaluation of our manuscript.

**Question 2**: Is the manuscript technically sound, and do the data support the conclusions?

Reviewer #3: Partly

Author Response: In light of this general feedback, and to ensure the manuscript is as robust and clear as possible, we have undertaken an additional comprehensive review of the entire manuscript.

**Question 3**: Has the statistical analysis been performed appropriately and rigorously?

Reviewer #3: Yes

**Question 4**: Have the authors made all data underlying the findings in their manuscript fully available?

Reviewer #3: Yes

**Question 5:** Is the manuscript presented in an intelligible fashion and written in standard English?

Reviewer #3: Yes

**Question 6:** Review Comments to the Author

Reviewer #3: (No Response)

Author Response: We thank Reviewer #3 for their positive responses (Question 3 – 6) which do not want further clarification and responses from us.

**Journal Requirements:** Please review your reference list to ensure that it is complete and correct. If you have cited papers that have been retracted, please include the rationale for doing so in the manuscript text, or remove these references and replace them with relevant current references. Any changes to the reference list should be mentioned in the rebuttal letter that accompanies your revised manuscript. If you need to cite a retracted article, indicate the article’s retracted status in the References list and also include a citation and full reference for the retraction notice.

Author Response: We have thoroughly reviewed our reference list for completeness and correctness. We confirm that no retracted articles are cited in the manuscript. All references have been updated to reflect the latest information, and their formatting adheres to PLOS ONE guidelines. No changes were necessary regarding retracted articles.

**Reviewer Specific comments**

**Comments**

The paper describes the effects of drinking mineral water on GI parasites. It is well composed and well organized paper. The language and coherence of the paper are commendable. However, there are flaws and issues of clarities in design, sampling technique, coprological methods, and results section as addressed below. Therefore, the paper needs revision and clarification before considering for publication.

**Author Response:** We sincerely thank the reviewer for their valuable time and constructive feedback on our manuscript. We have carefully addressed each specific comment in the revised manuscript, making clarifications and revisions as detailed in our point-by-point response.

**Materials and Methods**

**Reviewer Comment #1**: The sites selected for the study were all having access to mineral water (hora). To explore the effect of drinking mineral water on the GIT parasites burden or prevalence as indicated in the title and objectives, comparison sites, which do not have access to mineral water should be selected as control.

**Reviewer Comment #2**: The comparison was made within the same group, which have access to mineral water. This does not detect any correlation between mineral status and GIT burden

**Author Response to Reviewer Comment #1 & #2:** Thank you for your insightful comments regarding our study design and the absence of a control group (cattle without access to 'hora' mineral water). We acknowledge that such a design would be ideal for directly assessing the causal effect of 'hora' water consumption on gastrointestinal parasite (GIP) burden.

Our study, however, was specifically designed as an exploratory correlational analysis within a single population of zebu cattle that consistently accessed 'hora' mineral water. Our primary aim was to:

1. Characterize the mineral status and GIP burden in this specific population of cattle.
2. Establish correlations between varying individual serum mineral concentrations and GIP burden within this 'hora'-accessing group.
3. The results were compared against the standard parasite load acceptable to maintain normal animal health

We observed significant internal variation in both mineral concentrations and parasite burden among the sampled cattle. This internal variability allowed us to identify meaningful correlations, such as the strong negative associations between specific serum minerals and EPG, highlighting the potential role of mineral status in parasite resistance. The effect of Hora mineral water on GIT parasite load and serum minerals status was discussed in comparison with various findings and the standard range of values.

We have revised the manuscript's **title, objectives, and hypothesis** to more accurately reflect this exploratory correlational approach.

**Reviewer Comment #3:** It seems that the animals included in the study had low EPG (Table 3). It is difficult to discern if this low EPG is due to the effects of mineral water or lower parasite in the pasture in the absence of controls.

**Author Response:** We appreciate the reviewer's insightful observation regarding the relatively low EPG values. We acknowledge that, without a direct control group of cattle not accessing 'Hora' mineral water, it is challenging to definitively attribute the observed lower EPG solely to the mineral water's effects, as environmental factors could also contribute.

Our study, however, was designed as an exploratory correlational analysis, focusing on investigating the relationship between varying serum mineral concentrations and parasite burden within this specific population of zebu cattle routinely accessing 'Hora' mineral water.

Despite the absence of an external control, our study provides valuable internal evidence that sheds light on this point:

- **Plausible role of hora mineral water on EPG levels:** Our overall mean EPG of 161.87 and nematode EPG of 101.59 fall within the light to moderate infection range for grazing cattle. **The analysis of hora mineral water confirmed it contains adequate levels of essential minerals known to support robust immune function.** We therefore propose that the consistent access to these vital minerals via 'hora' water plausibly contributes to maintaining the observed lower to moderate EPGs in these cattle by enhancing their natural host resilience against parasitic infections.
- **Internal variability and correlations:** We observed significant internal variations in EPG and serum mineral status across districts, which strongly correlated with differences in 'Hora' access frequency. For instance, Borecha district, with higher serum mineral concentrations (likely due to more consistent hora access), exhibited lower EPGs, while Gechi district, with lower serum mineral levels, showed higher EPGs.
- **Strong correlational findings:** We found robust negative correlations between individual serum concentrations of key minerals (Fe, Mn, Cu, Zn, and Mo) and EPG, as well as a predictive negative binomial regression model. These findings consistently indicate that, within our study population, animals with better mineral status, potentially supported by hora mineral water intake, exhibited lower parasite loads. This suggests that adequate mineral nutrition helps mitigate GIP burden regardless of direct hora water effects alone.

Therefore, while acknowledging the limitations regarding definitive causality, our study's strength lies in identifying significant internal associations and gradients. These results strongly suggest a beneficial role for adequate mineral nutrition, consistently available from hora mineral water, in maintaining host resistance and mitigating GIP burden. We have further clarified this nuance and the study's correlational nature in the revised **discussion section**.

**Reviewer Comment #4:** It is better to use map to depict the study sites

**Author Response:** Thank you very much for your suggestion. The study area map was included in the revised manuscript.

**Reviewer Comment #5:**

**Sampling methods:**

**1. How did the authors randomize their selection? There is no individual animal identification system in Ethiopia, which is requisite for randomization.**

**Author Response:** Thank you very much for raising this important point regarding our sampling methodology. We acknowledge that the absence of a formal individual animal identification system in free-ranging cattle herds in Ethiopia presents a practical challenge for achieving true, classical randomization at the individual animal level. We agree that such systems are ideal for rigorous random selection.

However, to mitigate potential bias and ensure a selection process that was as close to random as practically feasible under these field conditions, we employed a systematic random sampling approach at the animal level within selected herds.

Specifically, within each of the 9 purposively selected herds (chosen based on consistent hora mineral water access):

1. **Herd Selection:** Herds were chosen based on their location near hora mineral water sources and the consistent access hora mineral water as mineral supplement.
2. **Animal Selection within Herds:** Upon arrival at a selected herd's grazing site, a predetermined number of animals (typically 20 animals per herd) meeting the inclusion criteria (Horro breed, sexually mature male or mature dry female, 5-7 years of age) were selected. To ensure a representative sample and minimize bias, we employed a systematic random sampling approach at the animal level within selected herds. This involved, selecting animals by pointing randomly to individuals within the grazing herd from a fixed vantage point, ensuring avoidance of conscious selection bias.

We have clarified this process in the **'Sample Collection'** section of the revised manuscript.

**Reviewer Comment #6:** The authors did not mention anything about the welfare issues in relation to handling of animals during sample collection (fecal and blood samples).

**Author Response:** We thank the reviewer for highlighting the critical importance of animal welfare during research procedures. While our manuscript stated that ethical clearance was obtained from Jimma University (RGS/588/2023) following EU guidelines (2010/63/EU) (**stated in manuscript under materials and methods section and the Approval letter was provided as supplementary information alongside with manuscript**), which inherently include welfare considerations in the approved protocol, we acknowledge that we did not explicitly describe the practical steps taken during sample collection. To address this, we have now added a statement to the **'Sample Collection'** section of the Materials and Methods, explicitly outlining the measures taken to ensure the welfare of the animals during fecal and blood sample collection.

**Reviewer Comment #7:**

**Coprological techniques:**

**1. How long did the fecal suspension sediment? This was not described by the authors**

**Author Response**: Thank you for your question regarding the sedimentation time for the fecal suspensions. We apologize if this detail was not sufficiently clear in our initial description. In our first round submitted manuscript, we indicated 10 minutes sedimentation time, and now we clarify the ambiguity in the **current revised manuscript and shown in yellow highlights under Coprological Examinations section**.

Upon review of standard protocols, we have revised our methodology to incorporate two sequential 5-minute sedimentation steps, which is a common practice for enhancing the concentration of trematode eggs. Specifically, after mixing 3g of fecal sample with 30 mL water and filtering, the suspension was allowed to sediment for an initial 5 minutes. The supernatant was then removed, the sediment was re-suspended in 5 mL of water, and allowed to sediment for a second 5-minute period before supernatant removal, staining, and microscopic examination. We have explicitly detailed these two 5-minute sedimentation steps in the revised manuscript to enhance clarity and reproducibility.

**Reviewer Comment #8:**

**2. Was quantitative analysis done for trematodes only? According to the description of the authors on page 5, it seems that a quantitative assay was done only for trematodes**

**Author Response**: Thank you for your question regarding the scope of our quantitative analysis. We appreciate the opportunity to clarify this point. Despite we conduct quantitative analysis for nematodes, cestodes, and trematodes, we missed to put all standard procedure in methodology section during manuscript preparation.

We clarified that the quantitative analysis using the modified McMaster technique was performed for the eggs of nematodes and cestodes, not trematodes.

For trematode eggs, which were identified qualitatively using the two-step sedimentation technique, a quantitative analysis was also performed using a Modified Stoll's dilution technique.

**Reviewer Comment #9:**

**Data analysis:**

The authors need to explicitly explain how the association between the EPG and serum mineral status was done using chi-squared test. Did they dichotomize the EPG and or the mineral concentrations? In the results section they reported Pearson correlation coefficient not Pearson Chi-square test.

**Authors Response:** We appreciate the reviewer's detailed query regarding our statistical analyses, which allows us to clarify the specific application of each test. We believe there might be a slight misunderstanding in the interpretation of our methodology:

- **Pearson Chi-squared Test**: The Pearson Chi-squared test was used exclusively to assess differences in GIP prevalence (i.e., the proportion of positive animals) among the study districts (as reported in Table 1). This test is appropriate for analyzing associations between categorical variables (e.g., district vs. GIP status: positive/negative). It was not used to assess the association between continuous EPG values and serum mineral concentrations. For this specific test, we did not dichotomize EPG or mineral concentrations, as it was applied to the binary outcome of GIP presence/absence per district.
- **Kruskal-Wallis Test and EPG/Serum Mineral Concentrations Across Districts (Table 2):** The χ2 reported in Table 2 corresponds to the Kruskal-Wallis H-statistic, which is a non-parametric alternative to one-way ANOVA. This test was correctly applied to analyze differences in the mean rank of continuous serum mineral concentrations and EPG counts across the categorical variable 'districts'. No dichotomization of EPG or mineral concentrations was performed for this analysis.
- **Association between EPG and Serum Mineral Status:** The primary method used to assess the relationship between continuous serum mineral concentrations and fecal egg counts (EPG) was Spearman's rank correlation. As stated in the methodology, non-parametric tests were employed because the distributions of serum mineral concentrations and fecal egg counts did not meet the assumptions of normality required for parametric tests. The results of these Spearman correlations are presented in Table 3.

Therefore, our study utilized Pearson's Chi-squared for prevalence comparisons, Spearman's rank correlation for initial continuous associations between EPG and minerals, and Negative Binomial Regression for in-depth modeling of EPG as a count outcome against continuous mineral concentrations. We confirm that Pearson correlation coefficients were not reported for the EPG-mineral association, as Spearman's correlation was the chosen non-parametric alternative. We have made a minor clarification in the **'Data Analysis'** section of the revised manuscript to explicitly emphasize the role of the Pearson Chi-squared test for prevalence differences, ensuring absolute clarity.

**Reviewer Comment #10:**

**Results:**

1. The authors reported the results to species level for parasites and to the genus level for others (figure 1). The authors did not perform culture. So, how did they do identification to species and genus level based flotation and sedimentation alone?

**Author Response:** We sincerely thank the reviewer for this crucial and insightful comment. We apologize for the oversight and lack of precision in our initial parasite identification reporting. The reviewer is absolutely correct: definitive differentiation of strongyle-type nematode eggs (e.g., Bunostomum spp., Cooperia spp., Marshallagia spp., Chabertia spp., and other Trichostrongyloids) to genus or species level is not reliably possible solely through flotation and sedimentation.

In the revised manuscript, we have corrected this by reclassifying all such eggs as 'strongyle-type nematode eggs' in the Results section and Figure 1. We confirm that other morphologically distinct eggs, specifically those of Fasciola spp., Paramphistomum spp., Moniezia spp., Trichuris spp., Nematodirus spp., Ascaris spp., and Strongyloides spp., possess unique characteristics that allow for their reliable identification to the genus level by microscopy.

We are truly grateful for this valuable feedback, which has significantly improved the accuracy and rigor of our manuscript.

**Reviewer Comment #11:**

2. The districts are dominantly found in mid to lowland agro ecology and similar weather conditions (from the study site description). Hence, how did the authors identify nematodes such as Nematodirus and Ostertagia? Their identification lacks reliability.

**Author Response:** Thank you for your comment and suggestion. As we committed in our previous response, we recognize that definitive differentiation of most strongyle-type nematode eggs to genus or species level (including those of Ostertagia spp.) based solely on morphology from flotation is not reliably possible. These eggs are morphologically very similar and typically require larval culture for precise identification. Therefore, in our revised manuscript, all such eggs, including any that might exhibit morphology vaguely consistent with Ostertagia or other undifferentiated strongyles, were consistently reported collectively as 'Strongyle-type eggs' throughout the text and in Figure 1. This grouping acknowledges the diagnostic limitations and ensures scientific accuracy, irrespective of the specific agro-ecological conditions.

Regarding Nematodirus spp., while these nematodes can be less prevalent in some lowland environments compared to cooler, temperate regions, their eggs are morphologically distinct from other strongyle-type eggs. Specifically, Nematodirus eggs are significantly larger, more elongated-oval, and typically possess a morula stage that fills the entire egg, making them readily distinguishable from other strongyle-type eggs. While Nematodirus is often associated with specific climatic conditions, its **presence in our samples was confirmed strictly based on these distinct morphological features.**

**Reviewer Comment #12:**

**Discussion:**

The authors need to discuss the significance their findings (provide explicit and implicit explanations) before making comparisons with previous observations

On page 10, the authors provided “The mean serum macro and micro mineral status in Zebu (Bos indicus) cattle grazing around and accessing hora mineral water might be associated with the presence of hora mineral water used as a supplement in the study areas” This explanation can be valid only if comparison is made with those cattle that graze away from hora and had lower concentrations.

**Author Response:** Thank you for your valuable feedback regarding the discussion of our findings, particularly your point on the need for clearer explanations of significance and the validity of inferring an association with Hora mineral water. We acknowledge that our initial phrasing may have implied a direct causal relationship that our study design, being an exploratory correlational study without an external control group, cannot definitively prove.

We agree that a direct comparison with cattle grazing away from Hora mineral water, showing lower concentrations, would indeed provide stronger evidence for Hora mineral water's direct impact on serum mineral status and parasite burden.

However, our discussion aimed to provide implicit and explicit explanations of our findings' significance by leveraging the internal variations observed within our study population, all of which had access to Hora mineral water.

We revised the discussion to clarify our inference. While we lacked a "no Hora water" control, we observed significant internal gradients within our study population. As noted in our previous responses, there were observable differences in the frequency and consistency of access and presumed consumption of Hora mineral water across the various sampled districts, even though all were considered to have access.

We observed that cattle from districts with more frequent and consistent access to Hora mineral water tended to exhibit higher serum mineral status and, significantly, also displayed lower gastrointestinal parasite burdens. Conversely, districts with less frequent Hora water access were associated with comparatively lower serum mineral levels and higher parasite burdens.

Therefore, our initial statement was an inference based on these observed internal correlations and gradients. This suggests that even within populations with access to Hora mineral water, the degree to which this resource is utilized and impacts the animal's mineral status can influence their susceptibility to parasites. This provides a nuanced understanding of the potential role of Hora mineral water as a contributing factor to herd health in the context of its natural availability.

We revised our discussion to elaborate on the significance of the observed correlations within our study population, introduce these crucial internal comparisons to provide a more robust and explicitly justified explanation for the potential association with Hora mineral water.
