# Supplementary material for: Association between serum mineral concentrations and gastrointestinal parasite burden in zebu cattle accessing ‘hora’ mineral water in southwestern Ethiopia
Source: PLoS One. 2025 Aug 18;20(8):e0329812. doi: 10.1371/journal.pone.0329812 (PMC12360600; doi:10.1371/journal.pone.0329812)
Supplement: Attachment — Submitted filename: Response to Reviewers.docx [file pone.0329812.s008.docx]

**Authors’ response to editor and reviewers’ comments**

Thank you for the opportunity to revise and resubmit our manuscript, “**Association between serum mineral concentrations and gastrointestinal parasite burden in zebu cattle accessing 'hora' mineral water in southwestern Ethiopia**" (PONE-D-25-01800R2). We appreciate the valuable feedback from the reviewers, which has significantly improved the quality of our manuscript. We have carefully addressed all comments raised by the academic editor and the reviewers, incorporating the suggested changes into the revised manuscript. A marked-up copy highlighting all revisions is provided as 'Revised Manuscript with Track Changes', and a clean version is provided as 'Manuscript(3)'.

In addition to addressing the specific comments below, we have also made a significant change to the presentation of our results. To enhance the clarity and immediate understanding of our key findings for the readers, we have modified and moved Table 4 and Table 5 from supporting information into the main body text of the manuscript. We believe that presenting this information directly within the main text provides a more comprehensive and accessible narrative of our research, allowing readers to readily grasp the nuances of our findings without needing to refer to supplementary files.

**Our detailed point-by-point responses to all comments are presented below.**

We really appreciate your help.

Ashenafi Miresa Kenea (corresponding author)

July 19, 2025

**Academic Editor Comments:**

The reviewers have completed their evaluation of your manuscript. I encourage you to revise and resubmit your work, ensuring that all reviewer comments are thoroughly addressed. Please incorporate the feedback carefully and provide a detailed, point-by-point response that clearly outlines every change made in response to the reviewers’ suggestions. In addition, kindly correct all typographical and grammatical errors, and ensure that the manuscript is prepared in full compliance with the journal’s formatting and submission guidelines. We look forward to receiving your revised submission.

**Author Response**: We thank the Academic Editor for this guidance. We have thoroughly reviewed our manuscript to ensure all previous reviewer comments are thoroughly addressed, as detailed in the previous revision and now in response to the current feedback. We have made a concerted effort to identify and correct all typographical and grammatical errors throughout the manuscript. We have also carefully checked and ensured full compliance with PLOS ONE’s formatting and submission guidelines, including the reference list.

**Reviewers' comments:**

**Reviewer's Responses to Questions**

**Comments to the Author**

1. If the authors have adequately addressed your comments raised in a previous round of review and you feel that this manuscript is now acceptable for publication, you may indicate that here to bypass the “Comments to the Author” section, enter your conflict of interest statement in the “Confidential to Editor” section, and submit your "Accept" recommendation.

**Reviewer #3: All comments have been addressed**

Author Response: We sincerely wish to thank Reviewer #3 for their time and his positive feedback. We are pleased to hear that all your comments have been addressed to your satisfaction.

**2. Is the manuscript technically sound, and do the data support the conclusions?**

The manuscript must describe a technically sound piece of scientific research with data that supports the conclusions. Experiments must have been conducted rigorously, with appropriate controls, replication, and sample sizes. The conclusions must be drawn appropriately based on the data presented.

**Reviewer #3: Yes**

Author Response: Thank you for your valuable feedback and for confirming the technical soundness of our manuscript and that our data support the conclusions. We appreciate your positive assessment.

**3. Has the statistical analysis been performed appropriately and rigorously?**

**Reviewer #3: Yes**

Author Response: Thank you for your valuable feedback and for confirming our statistical analysis was performed appropriately and rigorously. We appreciate your positive feedback.

**4. Have the authors made all data underlying the findings in their manuscript fully available?**

The PLOS Data policy requires authors to make all data underlying the findings described in their manuscript fully available without restriction, with rare exception (please refer to the Data Availability Statement in the manuscript PDF file). The data should be provided as part of the manuscript or its supporting information, or deposited to a public repository. For example, in addition to summary statistics, the data points behind means, medians and variance measures should be available. If there are restrictions on publicly sharing data—e.g. participant privacy or use of data from a third party—those must be specified.

**Reviewer #3: Yes**

Author Response: Thank you for your review and for confirming that we've made all underlying data fully available as per the PLOS Data Policy. We appreciate your positive assessment.

**5. Is the manuscript presented in an intelligible fashion and written in Standard English?**

PLOS ONE does not copyedit accepted manuscripts, so the language in submitted articles must be clear, correct, and unambiguous. Any typographical or grammatical errors should be corrected at revision, so please note any specific errors here.

Reviewer #3: Yes

Author Response: Thank you for your time to review and for confirming that our manuscript was presented in an intelligible fashion and written in standard English. We appreciate your positive assessment on this important aspect.

**6. Review Comments to the Author**

Please use the space provided to explain your answers to the questions above. You may also include additional comments for the author, including concerns about dual publication, research ethics, or publication ethics. (Please upload your review as an attachment if it exceeds 20,000 characters)

**Reviewer #3: Data analysis:**

Now the data analysis portion is well explained. One comment is that the authors said “Subsequently, a full multivariate model was constructed including all minerals as predictors, and stepwise regression using the Akaike Information Criterion (AIC) was then performed to select the most parsimonious model”. It is a multivariable model not multivariate model. Multivariate is when more than one outcome variable is modeled simultaneously but in this case it is more than one explanatory variables which are used to model one outcome variable. This needs to be corrected.

**Authors' Response:** We thank the reviewer for this important clarification regarding statistical terminology. We concur that since our analysis involved modeling a single outcome variable (EPG) with multiple explanatory (mineral) variables, the appropriate term is "**multivariable model**." We have corrected all instances of "multivariate model" to "**multivariable model**" in the methodology and results sections of the manuscript to ensure accuracy.

**Results:**
The districts are dominantly found in mid to lowland agro ecology and similar tropical weather conditions (from the study site description). Hence, how did the authors identify nematodes such as Nematodirus and Ostertagia? Their identification lacks reliability.

**Authors' Response:** Thank you for your valuable feedback regarding the identification of nematode eggs (Nematodirus and Ostertagia) given the agro-ecological setting. We agree that relying solely on egg morphology for definitive species identification in the Strongyle-type group can be limiting, especially given the morphological similarities among many nematode eggs.

While our initial identification was based on the extensive experience of our laboratory experts, we acknowledge that a more scientifically rigorous approach, such as larval culture or molecular techniques, is essential for absolute confirmation at the genus or species level. We recognize that even **highly experienced individuals can face challenges in differentiating between morphologically similar strongyle eggs.**

Upon reviewing your comments and aligning with current scientific consensus, we've revised our manuscript. We now report these morphologically similar eggs as "**Strongyle-type eggs**" throughout the text.

We have made the necessary changes throughout the manuscript to reflect this revised reporting. We apologize for any ambiguity or inaccuracy in our previous reporting and appreciate you guiding us toward this important correction.
